# Modifying Effect of Age on the Association between Ambient Ozone and Nighttime Primary Care Visits Due to Asthma Attack

**DOI:** 10.2188/jea.JE20081025

**Published:** 2009-05-05

**Authors:** Shin Yamazaki, Masayuki Shima, Michiko Ando, Hiroshi Nitta

**Affiliations:** 1 Department of Epidemiology and Healthcare Research, Graduate School of Medicine and Public Health, Kyoto University, Kyoto, Japan; 2 Department of Public Health, Hyogo College of Medicine, Nishinomiya, Hyogo, Japan; 3 Department of Respirology, Graduate School of Medicine, Chiba University, Chiba, Japan; 4 Environmental Health Science Division, National Institute for Environmental Studies, Tsukuba, Ibaraki, Japan

**Keywords:** air pollution, asthma, ozone, particulate matter, preschool children

## Abstract

**Background:**

We examined the association between short-term exposure to outdoor air pollution and nighttime primary care visits due to asthma attack. We also investigated the modifying effects of age on this association.

**Methods:**

A case–crossover study was conducted at a primary care clinic in metropolitan Tokyo. The subjects were 308 children aged 0–14 years and 95 adolescents and adults aged 15–64 years. All subjects made visits to the clinic for an asthma attack at between 7 PM and 12 AM. Data on hourly concentrations of particulate matter with a 50% cut-off aerodynamic diameter ≤2.5 µm (PM_2.5_), ozone, and nitrogen dioxide (NO_2_) were obtained. A conditional logistic regression model was used to estimate odds ratios (ORs) of primary care visits per unit increment of each air pollutant.

**Results:**

Among children, the ORs in warmer months per 10 ppb increment of the 24-hour mean concentration of ozone were 1.16 (95% confidential interval [CI], 1.00–1.33) adjusted for temperature, and 1.29 (95% CI, 1.08–1.55) adjusted for PM_2.5_, NO_2_, and temperature. With respect to modification of the association by age, the ORs for 24-hour mean concentration of ozone—after adjustment for PM_2.5_, NO_2_ and temperature in warmer months—in children aged 0–1 years, 2–5 years, and 6–14 years were 1.06 (95% CI, 0.63–1.78), 1.37 (95% CI, 1.05–1.71), and 1.25 (95% CI, 0.87–1.82), respectively. There was no association between ozone and primary care visits among adults.

**Conclusions:**

An association was found between ozone and nighttime primary care visits for asthma attack in warmer months; the association was greater among preschool children.

## INTRODUCTION

Children’s exposure to air pollution is a special concern because their immune system and lungs are not fully developed. Children spend more time outside, where levels of pollution from traffic, power plants, and other sources are generally higher. Exposure to ambient air pollution, including particulate matter (PM), ozone (O_3_), and nitrogen dioxide (NO_2_), is associated with many adverse health outcomes ranging from increased symptoms of allergic airway disease to increased mortality.^1^^–^^3^ Children are considered to be more sensitive to air pollution than adults,^4^ and asthmatic children are particularly vulnerable to the adverse health effects of air pollution. Studies of asthmatic children conclude that exposure to high concentrations of O_3_ or PM significantly increases the risk of respiratory symptoms and asthma medication use, and diminishes lung functions such as peak expiratory flow rate and forced expiratory flow rate.^5^^–^^9^ However, among children, age-related sensitivity to air pollutants and adverse respiratory effects has been investigated in only a small number of studies. Babin et al^10^ observed an association between pediatric emergency room visits for asthma exacerbations and outdoor O_3_, especially in children aged 5 to 12 years; however, they noted no such association among children aged 1 to 4 years.

To investigate the associations between short-term exposure to air pollution and adverse pulmonary outcomes—as well as the modifying effects of age—we collected and analyzed hourly air-pollution data and the records of nighttime primary care visits due to asthma attack.

## METHODS

### Subjects

The setting of this study was the Ichikawa Emergency Clinic in Ichikawa, Japan. The clinic offers emergency care between 7 PM and 12 AM (midnight) on weekdays and Saturdays, and between 10 AM and 12 AM on Sundays and national holidays. Ichikawa City borders Tokyo Bay and is located in the Tokyo metropolitan area, approximately 20 km from central Tokyo. The area of the city is 56 km^2^, and its population is 470 000. The subjects were patients aged 0 to 64 years who presented with an asthma attack, lived in Ichikawa City, and visited the municipal emergency primary care clinic between 7 PM and 12 AM from September 1, 2002 through August 31, 2003. We limited the study time to between 7 PM and 12 AM in order to examine the association between daytime exposure and adverse health effects occurring at night. We excluded patients who visited the clinic on national holidays, for reasons discussed in the section on statistical analysis. The medical records of all patients were reviewed retrospectively, and subject age, sex, diagnosis, treatment, medication, and date and time of visits were recorded. Eligible subjects were patients that had paroxysmal dyspnea and were diagnosed with asthma by their primary care physician, and for whom any type of bronchodilator was prescribed. We obtained approval from the Ichikawa local government to use personal identifiable information from the municipal clinic.

### Air pollutants

Data on hourly concentrations of NO_2_ and O_3_ from September 2002 through August 2003 were obtained from the Ichikawa local government. The monitoring station where these air pollutants were measured was located on a residential street in the city. In addition, we measured hourly concentrations of particulate matter with a 50% cut-off aerodynamic diameter ≤2.5 µm (PM_2.5_) using the R&P TEOM-1400 (Rupprecht & Patashnick Co, Inc, Albany, NY) at a location near the monitoring station, from September 2002 through August 2003.

### Statistical analysis

We used time-stratified case–crossover analysis, a technique for assessing brief changes in risk associated with transient exposures.^11^^,^^12^ Case–crossover analyses require exposure data for cases only, and are regarded as a special type of case–control study in which each case serves as his or her own control. This design has the advantage of controlling for potential confounding caused by fixed individual characteristics, such as sex, race, diet, and age. “Time-stratified” indicates the method by which the control periods were chosen. Specifically, we stratified time into months to select days for control periods that fell on the same day of the week within the same month as the date of the primary care visit (day of the index period). For example, if a nighttime primary care visit due to asthma attack were to occur on September 18, the 3 control days would be September 4, 11, and 25. The control periods were also matched by time of index periods. Therefore, this approach also controls for long-term trends, seasonality, day of the week, and circadian rhythms. The merits of case–crossover designs in studies of the health effects of air pollution have been discussed in detail by Schwartz.^13^

We excluded patients who visited the clinic on national holidays because of bias in control selection. That is, if patients whose visits occurred on holidays were included as subjects, the estimated relative risks would be lower than expected because the concentrations of air pollutants on holidays (days for the index periods) were usually, and systematically, lower than on non-holidays (days for control periods).

In our analysis we first converted the hourly air pollution data into 6-hour mean concentrations, and we examined the associations between the 6-hour mean concentrations of each air pollutant and the risk of nighttime primary care visits due to asthma attack. These concentrations were subject-specific values averaged over the 6 hours before the index time. Exposure to air pollutants during the period from 6 hours before a nighttime primary care visit due to asthma attack to the hour of the visit was defined as exposure with a time lag of 0 to 6 hours (lag 0–6), exposure during the period 12 hours before to 6 hours before was defined as exposure with a lag of 6 to 12 hours (lag 6–12), etc. Lagged-hour exposures up to lag 18–24 were examined. We estimated odds ratios (ORs) of nighttime primary care visits due to asthma attack per 10 µg/m^3^ difference in PM_2.5_ in a single-pollutant model adjusted for 6-hour mean temperature. In like manner, we also estimated ORs of primary care visits per 10 ppb difference in NO_2_, and per 10 ppb difference in O_3_. We also estimated ORs of nighttime primary care visits due to asthma attack per 10 µg/m^3^ difference in PM_2.5_, per 10 ppb difference in NO_2_, and per 10 ppb difference in O_3_ in a multipollutant model adjusted for 6-hour mean temperature.

Second, we converted the hourly air pollution data into 24-hour mean concentrations and examined the effect of the 24-hour mean concentration of air pollutants (lag 0–24) on nighttime primary care visits due to asthma attack. We estimated ORs of nighttime primary care visits due to asthma attack per 10 µg/m^3^ difference in 24-hour mean PM_2.5_, per 10 ppb difference in NO_2_, and per 10 ppb difference in 24-hour mean O_3_ in both a single-pollutant model and a multipollutant model adjusted for 24-hour mean temperature.

Third, we examined the effect of the daytime 8-hour mean concentrations of air pollutants in the period from 8 AM through 4 PM on nighttime primary care visits due to asthma attack. We estimated ORs of nighttime primary care visits due to asthma attack per 10 µg/m^3^ difference in 8-hour mean PM_2.5_, per 10 ppb difference in 8-hour mean NO_2_, and per 10 ppb difference in 8-hour mean O_3_ in both a single-pollutant model and a multipollutant model adjusted for 8-hour mean temperature.

To examine the modifying effects of age on the association between air pollutants and nighttime primary care visits due to asthma attack, the data were grouped into these patient age groups: 0 to 1 year, 2 to 5 years, 6 to 14 years, 0 to 14 years and 15 to 64 years. The aforementioned analyses were performed independently for each of these groups. Moreover, all models took into consideration the effects of seasonality and those due to unusually high and low temperatures: modified effects were examined by using a 2-level indicator variable for the warmer months (April through September) and the colder months (October through March).

The PHREG procedures of SAS release 8.2, SAS Institute, Inc, Cary, NC, USA were used to perform the conditional logistic regression. All tests were 2-tailed, and alpha was set at 0.05. We computed ORs and their 95% confidence intervals (CIs). In this study we used several test procedures; therefore, multiple testing issues arose. However, we elected not to devise a countermeasure to these multiple testing issues because we felt that it was more important to identify all possible elevated risks due to air pollutants.

## RESULTS

**Figure 1.**
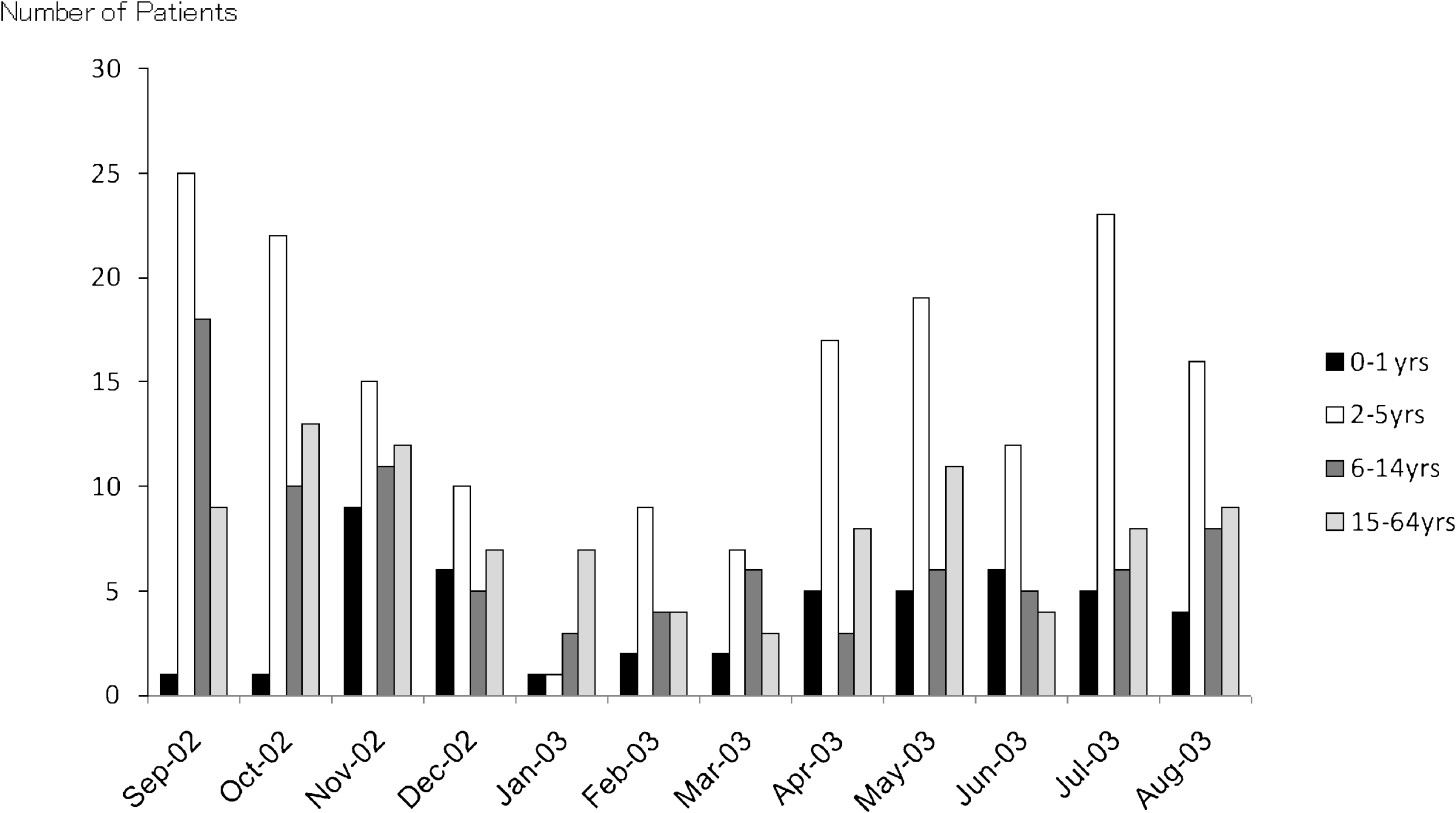
Number of patients who visited the Ichikawa Emergency Clinic due to asthma attack between 7 PM and 12 AM, by month, from September 1, 2002 to August 31, 2003

The characteristics of the subjects are shown in Table 1. Among the 403 subjects, 308 were children aged 0 to 14 years and 95 were adolescents/adults aged 15 to 64 years. Among the 308 children, 47 were younger than 2 years, 176 were preschool children aged 2 to 5 years, and 85 were school children aged 6 to 14 years. Figure 1 shows the number of cases in each month of the study period.

**Table 1.** Age and sex of subjects

Age group (years)	Sex		Total

	Male	Female	
0–1	26	20	47
2–5	110	66	176
6–14	66	18	85
15–64	55	40	95
Total	257	144	403*

*Data on sex for 2 subjects were missing.

The hourly mean concentrations of air pollutants are shown in Table 2. The mean concentration of O_3_ in warmer months was higher than in colder months. However, the mean concentration of NO_2_ in warmer months was lower than in colder months. Table 3 shows the correlation coefficients of hourly measured concentrations for the warmer months and colder months from September 2002 through August 2003.

**Table 2.** Summary of hourly concentrations of air pollutants

		Total hours measured	Mean	Standard deviation	Maximum
Warmer months (April through September)					
​ PM_2.5_	µg/m^3^	4159	18.6	(11.4)	221.4
​ NO_2_	ppb	4366	16.9	(11.8)	77.0
​ O_3_	ppb	4359	33.7	(22.9)	224.0
Colder months (October through March)					
​ PM_2.5_	µg/m^3^	4360	19.6	(15.2)	112.4
​ NO_2_	ppb	4276	27.2	(16.4)	107.0
​ O_3_	ppb	4257	22.5	(18.5)	113.0

PM_2.5_: particulate matter with a 50% cut-off aerodynamic diameter ≤2.5 µm; NO_2_: nitrogen dioxide; O_3_: ozone.

**Table 3.** Correlation coefficients between hourly measured concentration of PM_2.5_, ozone (O_3_), nitrogen dioxide (NO_2_), and temperature

	NO_2_	O_3_	Temperature
Warmer months (April through September)			
​ PM_2.5_	0.40	0.18	0.09
​ NO_2_		−0.44	0.03
​ O_3_			0.08
Colder months (October through March)			
​ PM_2.5_	0.65	−0.41	0.11
​ NO_2_		−0.72	−0.17
​ O_3_			0.30

PM_2.5_: particulate matter with a 50% cut-off aerodynamic diameter ≤2.5 µm; NO_2_: nitrogen dioxide; O_3_: ozone.

### The effect of O_3_ on primary care visits due to asthma attack among children and adolescents/adults

**Figure 2.**
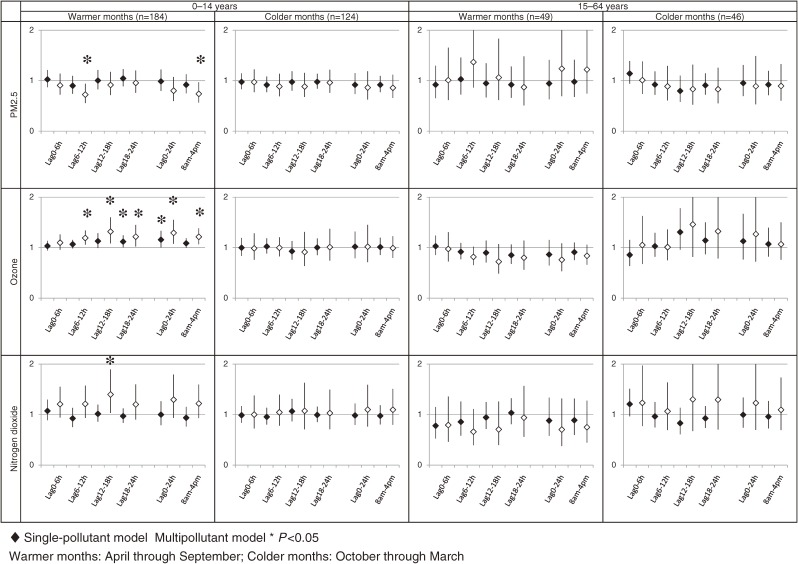
Associations between air pollutants and nighttime primary care visits due to asthma attack, by season and age. The associations are shown as odds ratios and their 95% confidence intervals per unit increment of each pollutant. The unit increments were 10 µg/m^3^ for PM_2.5_, 10 ppb for ozone, and 10 ppb for nitrogen dioxide

Figure 2 shows the association between nighttime primary care visits due to asthma attack among subgroups of children aged 0 to 14 years and adolescents/adults aged 15 to 64 years, stratified by warmer months and colder months. Among children, the ORs of nighttime primary care visits due to asthma attack per 10 ppb increment of O_3_ at time lag 6–12, lag 12–18, and lag 18–24 in the single-pollutant model were 1.07 (95% confidential interval [CI], 0.99–1.15), 1.13 (95% CI, 0.99–1.29), and 1.12 (95% CI, 1.01–1.24), respectively. When using the multipollutant model, the ORs were 1.19 (95% CI, 1.06–1.34), 1.32 (95% CI, 1.08–1.60), and 1.22 (95% CI, 1.02–1.45), respectively. The 24-hour mean concentration of O_3_ was also associated with nighttime primary care visits due to asthma attack in both the single-pollutant model (OR = 1.16; 95% CI, 1.00–1.33) and multipollutant model (OR = 1.29; 95% CI, 1.08–1.55). In addition, the daytime 8-hour mean concentration of O_3_ was also associated with nighttime primary care visits due to asthma attack. The ORs in the single-pollutant model and multipollutant model were 1.09 (95% CI, 0.99–1.19) and 1.21 (95% CI, 1.07–1.38), respectively. We found no association between O_3_ and nighttime primary care visits due to asthma attack in colder months.

### The effect of PM_2.5_/NO_2_ on primary care visits due to asthma attack among children and adolescents/adults

With respect to PM_2.5_, we observed an association between nighttime primary care visits due to asthma attack and PM_2.5_ among children in warmer months at lag 6–12, and in daytime 8-hour mean concentration among children, but not adults. With respect to NO_2_, we found an association between nighttime primary care visits due to asthma attack and NO_2_ in warmer months at lag 12–18 among children, but not adults.

### Association between O_3_ and primary care visits due to asthma attack among children in warmer months, by age subgroup

**Figure 3.**
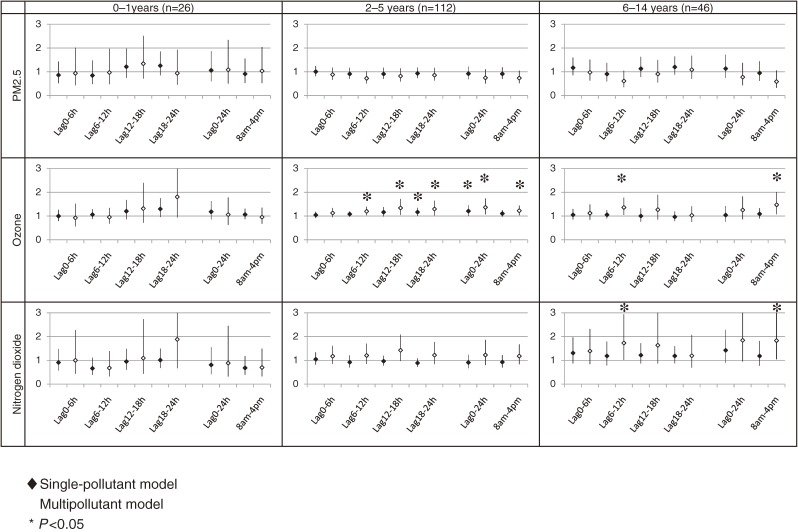
Associations between air pollutants and nighttime primary care visits due to asthma attack in warmer months (April through September), by age. The associations are shown as odds ratios and their 95% confidence intervals per unit increment of each pollutant. The unit increments were 10 µg/m^3^ for PM_2.5_, 10 ppb for ozone, and 10 ppb for nitrogen dioxide

Figure 3 shows the results of an age-stratified subgroup analysis among children in warmer months. With respect to the association between O_3_ and nighttime primary care visits due to asthma attack in the 2- to 5-year-old subgroup, the ORs of nighttime primary care visits due to asthma attack at time lag 6–12, lag 12–18, and lag 18–24 in the single-pollutant model were 1.08 (95% CI, 0.97–1.20), 1.16 (95% CI, 0.98–1.37), and 1.16 (95% CI, 1.01–1.34), respectively. When using the multipollutant model, the ORs were 1.20 (95% CI, 1.03–1.39), 1.34 (95% CI, 1.05–1.71), and 1.30 (95% CI, 1.02–1.64), respectively. The 24-hour mean concentration of O_3_ was also associated with nighttime primary care visits due to asthma attack. ORs were 1.20 (95% CI, 1.00–1.45) in the single-pollutant model and 1.37 (95% CI, 1.08–1.73) in the multipollutant model. In addition, daytime 8-hour mean concentration of O_3_ was also associated with nighttime primary care visits due to asthma attack. The ORs in the single-pollutant model and in the multipollutant model were 1.10 (95% CI, 0.98–1.24) and 1.22 (95% CI, 1.04–1.44), respectively. Among children aged 6 to 14 years, we noted elevated ORs for nighttime primary care visits due to asthma attack at lag 6–12 and in 24-hour mean concentration of O_3_, using a multipollutant model; the ORs were 1.36 (95% CI, 1.05–1.77) and 1.47 (95% CI, 1.08–2.01), respectively. No other associations were observed. For example, the ORs of nighttime primary care visits due to asthma attack for 24-hour mean concentration of O_3_ using a multipollutant model among children 0 to 4 years and 6 to 14 years were 1.06 (95% CI, 0.63–1.78) and 1.27 (95% CI, 0.88–1.84), respectively.

### Association between PM_2.5_/NO_2_ and primary care visits due to asthma attack among children in warmer months, by age subgroup

With respect to NO_2_, we observed an association among children aged 6 to 14. The ORs of nighttime primary care visits due to asthma attack at time lag 6–12, and the daytime 8-hour mean concentration of NO_2_ in a single-pollutant model, were 1.73 (95% CI, 1.02–2.93) and 1.83 (95% CI, 1.05–3.20), respectively. With respect to PM_2.5_, we found no associations among the various age groups with decreased ORs.

### Association between air pollutants and primary care visits due to asthma attack among children in colder months, by age subgroup

**Figure 4.**
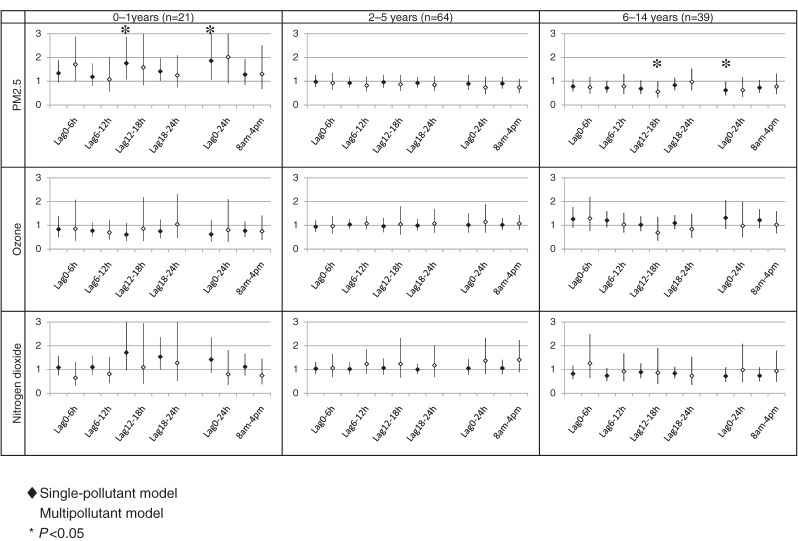
Associations between air pollutants and nighttime primary care visits due to asthma attack in colder months (October through March), by age. The associations are shown as odds ratios and their 95% confidence intervals per unit increment of each pollutant. The unit increments were 10 µg/m^3^ for PM_2.5_, 10 ppb for ozone, and 10 ppb for nitrogen dioxide

Figure 4 shows the results of an age-stratified subgroup analysis of children in colder months. With respect to the association between PM_2.5_ and nighttime primary care visits due to asthma attack in colder months among children aged 0 to 1 years, we observed slightly higher ORs at various time lags within 1 day. For example, the ORs of nighttime primary care visits due to asthma attack for 24-hour mean concentration of PM_2.5_ in single-pollutant and multipollutant models were 1.86 (95% CI, 1.06–3.27) and 2.02 (0.92–4.41), respectively. However, among children aged 6 to 14 years, we found a significant inverse association between PM_2.5_ and nighttime primary care visits due to asthma attack in colder months: the ORs of nighttime primary care visits due to asthma attack at lag 12–18 using a multipollutant model, and for 24-hour mean concentration of PM_2.5_ using a single-pollutant model, were 0.56 (95% CI, 0.31–1.00) and 0.62 (95% CI, 0.40–0.98), respectively.

## DISCUSSION

We found an association between O_3_ and nighttime primary care visits due to asthma attack among children aged 0 to 14 years—especially those aged 2 to 5 years—in warmer months. We also found an association between PM_2.5_ and primary care visits among children 0 to 14 years in warmer months (resulting in decreased ORs), among children 0 to 1 years in colder months (resulting in increased ORs), and among children 6 to 14 years in colder months (resulting in decreased ORs). Moreover, we found an association between NO_2_ and primary care visits in warmer months among children 0 to 14 years (resulting in increased ORs).

### O_3_ and primary care visits due to asthma attack

The association we observed between O_3_ and visits due to asthma attack was consistent with previous studies. A recent US Environmental Protection Agency analysis of ambient O_3_ health effects concluded that children with asthma suffer acute adverse health consequences at current ambient levels of O_3_.^3^ Studies of these adverse outcomes have examined asthma-related hospital discharges, but have yielded some of the least consistent data. Babin et al^10^ and Moore et al^14^ observed an association between pediatric emergency room visits for asthma exacerbations and outdoor O_3_.

In the present study, the association among preschool children aged 2 to 5 years was stronger than that among school children aged 6 to 14 years. One reason for this finding may be that the upper and lower airways of preschool children are at an early stage of development, and are thus shorter than in school children. In addition, Babin et al^10^ speculated that younger children have higher alveolar ventilation relative to their body mass, as well as a higher peripheral airway resistance, which results in younger children having a greater risk of adverse ventilator effects. However, it is important to note that the association between O_3_ and nighttime primary care visits due to asthma attack in infants aged 0 to 1 years could be affected by misestimation of levels of exposure to O_3_. Infants might spend more time in their homes, so calculations of exposure levels based on concentrations of outdoor air pollutants would be less accurate than those for preschool children/students, who spend a larger amount of time outside. Moreover, respiratory diseases other than asthma might be misclassified in infants because of the difficulty in diagnosing the respiratory symptoms of asthma in this age group. Therefore, an association between O_3_ and nighttime primary care visits due to asthma attack might be more likely to be observed among preschool children aged 2 to 5 years in warmer months.

Why was the strong association between O_3_ and primary care visits found only during the warmer months? First, as mentioned above, in colder months, misestimation of exposures to air pollutants might be larger because we were unable to measure the effects of indoor space heaters on the concentrations of air pollutants. Second, people spend more time outdoors and windows are left open longer during warmer months, so indoor concentrations are closer to those found outdoors. Third, the mean concentration of O_3_ in warmer months was 1.5 times higher than that in colder months.

Some associations that were found in the multipollutant model were not observed in the single-pollutant model. For example, the increased ORs for higher O_3_ were found at lag 6–12 among subjects aged 6 to 14 years in warmer months. Higher ORs for increased NO_2_ were also found at lag 6–12 among subjects aged 6 to 14 years in warmer months. However, the reasons for these findings remain unclear. O_3_ is formed by the action of short wavelength solar radiation on NO_2_. We observed an inverse relation between O_3_ and NO_2_: the correlation coefficient was −0.44 in warmer months (Table 3).

### PM_2.5_ and primary care visits due to asthma attack

The associations between air pollutants other than PM_2.5_ and primary care visits were not consistent. That is, the ORs among infants aged 0 to 1 years were increased, but the ORs among preschool children aged 2 to 5 years and school children aged 6 to 14 years were decreased. We believe that the association between PM_2.5_ and nighttime primary care visits due to asthma attack in subjects aged 0 to 1 years may be a chance result for 3 reasons. First, the sample size of this group was small (*n* = 21). Second, subjects with respiratory diseases other than asthma might have been included in this age group, because it is difficult to diagnose the respiratory symptoms of asthma in infants. Third, in colder months, misestimation of exposure to PM_2.5_ might be larger because we were unable to measure the effect of indoor space heaters on the concentrations of air pollutants. As mentioned above, infants would spend more time in their homes than preschool children or older students. However we cannot explain why the ORs among preschool children and school children were decreased.

Although other studies have shown significant associations between particulates and visits due to asthma-related symptoms,^15^^–^^17^ Fusco et al^18^ and Babin et al^10^ found no significant association between pediatric emergency room visits due to asthma-related symptoms and particulates. These contradictory results may be due to different particulate size distributions and concentrations at different locations, different types of pollution sources at those locations, and different periods of investigation. Several epidemiologic studies have shown that the oxidant properties of ambient air contribute to adverse health outcomes. Romieu et al^19^ studied the effects of sulfur dioxide, PM_10_, NO_2_, and O_3_ on asthmatics in Mexico City and noted that O_3_ was most closely associated with decrements in lung function in children.

### Time lag

In this study, we assessed nighttime primary care visits due to asthma attack with respect to elevated 6-hour mean concentration of air pollution at various lag times. O_3_ is a powerful oxidant, and it reacts with a wide range of cellular components and biological materials. Both experimental and epidemiologic studies have shown short-term reversible deficits in lung function resulting from O_3_. These deficits persist for a period ranging from hours to days. However, there would be various time lags between elevated concentrations of air pollutants and asthma exacerbations, and there would also be various time lags between asthma exacerbations and the time of nighttime primary care visits due to asthma attack. We found no association between nighttime primary care visits due to asthma attack and air pollution at lag 0–6. Therefore, we speculate that although the adverse health effects of O_3_ might appear immediately, presentation for medical evaluation might occur somewhat later.

### Limitations

The results of this study should be evaluated with caution for 4 reasons. First, the significance of the association between air pollution and nighttime primary care visits due to asthma attack is diminished because primary care visits due to asthma attack are only a surrogate measurement for asthma exacerbations. There would be various time lags between elevated concentrations of an air pollutant and asthma exacerbations, and there would also be various time lags between asthma exacerbations and the time of a primary care visit. These variations in time lags would affect statistical associations between air pollutants and nighttime primary care visits due to asthma attack. Second, we were unable to measure the effect of indoor space heaters on the concentrations of air pollutants. In Japan, indoor space heaters are commonly used in winter, especially in the period from December through February. Therefore, we believe that the effect of indoor space heaters on the association between the concentration of O_3_ and nighttime primary care visits due to asthma attack in warmer months was small. Third, ambient concentrations of air pollutants might act as surrogate measures of exposure to other agents or to specific pollution sources that are in fact responsible for the observed association between O_3_ and nighttime primary care visits due to asthma attack. Moreover, although we describe our results as O_3_-related effects, O_3_ is likely to be the best representative of the pollutants available for analysis of the gaseous oxidant species produced by complex photochemistry. Fourth, selection of subjects for this study may have been subject to problems of external validity because we restricted the subjects to nighttime patients.

### Conclusion

We found an association between O_3_ and nighttime primary care visits due to asthma attack among pediatric patients—especially among preschool children aged 2 to 5 years—in warmer months. This study provides additional support for the present regulatory position, which maintains that regional air quality needs to be modified in consideration of the high sensitivity of children to air pollution.
